# The political economy of digital profiteering: communication resource mobilization by anti-vaccination actors

**DOI:** 10.1093/joc/jqac043

**Published:** 2022-12-24

**Authors:** Aliaksandr Herasimenka, Yung Au, Anna George, Kate Joynes-Burgess, Aleksi Knuutila, Jonathan Bright, Philip N Howard

**Affiliations:** University of Oxford, UK; University of Oxford, UK; University of Oxford, UK; University of Oxford, UK; University of Oxford, UK; University of Oxford, UK; The Alan Turing Institute, UK; University of Oxford, UK

**Keywords:** hybrid media, vaccines, COVID-19, misinformation, communication resource mobilization

## Abstract

Contemporary communication requires both a supply of content and a digital information infrastructure. Modern campaigns of misinformation are especially dependent on that back-end infrastructure for tracking and targeting a sympathetic audience and generating revenue that can sustain the campaign financially—if not enable profiteering. However, little is known about the political economy of misinformation, particularly those campaigns spreading misleading or harmful content about public health guidelines and vaccination programs. To understand the political economy of health misinformation, we analyze the content and infrastructure networks of 59 groups involved in communicating misinformation about vaccination programs. With a unique collection of tracker and communication infrastructure data, we demonstrate how the political economy of misinformation depends on platform monetization infrastructures. We offer a theory of communication resource mobilization that advances understanding of the communicative context, organizational interactions, and political outcomes of misinformation production.

## Introduction

Digital misinformation campaigns are obscure and able to conceal many of the mechanisms that allow them to function. Some of these mechanisms are at the center of the economy of online misinformation—how and by whom misinformation campaigns are funded, what infrastructure they use to sustain themselves materially, or whether they manage to make any profit (Freelon & Wells, 2020; Howard, 2020). Interest in the political economy of online misinformation has recently been growing, as evidenced by the studies of Edwards (2021), Han (2015), and Ong and Cabañes (2019). Our article follows on from these empirically focused studies. It contributes to the communication theory by discussing the communicative context, organizational interactions, and political outcomes of misinformation production, thus complicating the understanding of the infrastructural and organizational features of digital propaganda. We analyze how issue-oriented misinformation actors—in our case, groups that spread misleading anti-vaccination information—gather material resources, such as money or web infrastructure. We examine the multiple ways these actors aggregate these resources as part of their communication strategies.

Research into the political economy of misinformation is rare, despite growing evidence that commercial motives and profiteering drives its production. We still know relatively little about external systems, reward structures, and power centers that supply online misinformation. On the one hand, the communication history of propaganda highlights elite-driven nature of large-scale pre-digital campaigns (Chadwick & Stanyer, 2022). Advertising and public relations, historically “the most pervasive form of propaganda” (Jowett & O′Donnell, 2011, p. 105), seems to be the craft of political and economic elites. On the other hand, in the most recent research on misinformation, the role of advertising and public relations has been “largely overlooked,” though misinformation is a “well-established tool in public relations” where falsehoods are “organised” (Edwards, 2021, p. 168). Our expectation that large communications firms and media elites might produce misinformation has not been recently interrogated, and scholarship has not devoted much attention to the production side of misinformation and the infrastructural resources required for production. For example, none of the 58 indicators featured in a recent comprehensive summary of variables that are used in misinformation literature (Chadwick & Stanyer, 2022) concern the material resources of misinformation production.

Our study addresses this limitation and offers an economy-focused theoretical framework that can be integrated into other relevant theories (Chadwick & Stanyer, 2022; Ong & Cabañes, 2019; Reese & Chen, 2022). For some people involved in the infrastructures of misinformation, profit or material gain may be the primary motivation for the content they produce. A number of recent studies point out that these people are driven financially but also politically and psychologically (Ong & Cabañes, 2019). Their pay structure can emphasize quantity over quality; their rigid work arrangements can lead to failed outcomes in misinformation operations (Au et al., 2020, Han, 2015). For others, material resources may simply help sustain their activities. Hence, some reports have recommended demonetization as a strategy to tackle the problem of misinformation (Center for Countering Digital Hate, 2020). However, due to the limited availability of scientific research in this area, there is little systematic evidence on exactly how misinformation actors monetize their operations. Specifically, how do anti-vaccination groups leverage their content and develop their infrastructure for misinformation production?

Anti-vaccination groups can be harmful given the benefits vaccinations have brought society. Vaccines are a vital component of contemporary public health strategies. They play a critical role in reducing (or even eradicating) disease and have saved millions of lives worldwide (Leach & Fairhead, 2007). Despite these benefits, the belief that vaccinations are unsafe and unnecessary persists and has proven resurgent at critical moments, overemphasizing the risk of side effects (Bandari et al., 2017; Kearney et al., 2019; Kennedy, 2019; Larson, 2018). Such beliefs are among the primary reasons for refusing to take vaccines, including a COVID-19 vaccine, an issue of urgent public health concern. At the time of writing, only 80% of the UK population had taken a Covid-19 vaccine, while in France one in three people did not agree that vaccines are safe (Wellcome Trust & Gallup, 2019). The phenomenon of vaccine hesitancy has been with us as long as vaccination itself, but the causes of vaccine hesitancy vary by context and vaccination type (Leach & Fairhead, 2007; Poland & Jacobson, 2011). Scholarship makes a distinction between vaccine-hesitant individuals weighing “legitimate doubts and concerns” and committed “activism against any form of vaccination” (Dubé et al., 2021). Recent attention has focused on how the latter organized anti-vaccination groups on social media (Bandari et al., 2017; Larson, 2020). Some of them have seen their profile boosted by the pandemic through promoting narratives that vaccines have more widespread severe side effects than reported.

These “anti-vaxxers,” as they are often known, are vocal and distributed across the Internet, hence able to achieve a kind of over-representation (Kearney et al., 2019; Motta et al., 2018). The Center for Countering Digital Hate (2020) argued that on Facebook alone at least 31 million users were part of anti-vaccine groups. Along with a vibrant social media presence, these actors operate websites that emphasize parents’ right to decide if their child receives a vaccine, arguing that public health institutions violate parents’ rights when vaccinations become mandatory (Elliman & Bedford, 2013). Anti-vaccination websites often share content on the dangers of vaccines and challenge scientific facts with alternative narratives (Kata, 2012).

Exposure to this information has been linked to growing vaccine hesitancy. For example, Ford and Alwan’s (2018) research into pregnant women in the UK found that those who reported using social media to research antenatal vaccinations were 58% less likely to accept the whooping cough vaccine while pregnant. A US study found a “significant relationship” between parents who described social media as their primary source of information and a propensity not to immunize their children (Wachob & Boldy, 2019). Earlier, a UK study found parents who “used the Internet to obtain information” about immunization were “significantly more likely to delay or refuse vaccination,” while those who visited chat rooms or discussion forums were “particularly likely to decline vaccination” (Campbell et al., 2017). During the COVID-19 pandemic, vaccination was linked to a broader array of health misinformation circulating on social media. Around a third of respondents to a six-country survey reported that they had seen “a lot or a great deal of false or misleading” information about COVID-19 on social media during the previous week (Nielsen et al., 2020). These studies demonstrate that vaccine hesitancy on the Internet is a pressing problem, and one that is linked to misinformation actors.

To outline our analysis of the political economy of issue-oriented misinformation, our article takes the following structure. First, we set out a framework for studying how misleading or harmful content can be used to extract resources that distinguishes between three main strategies: radical social movements, online celebrities, and junk news. On this basis, we draw out a typology of such approaches. We argue that the existing literature does not fully capture the hybrid approaches adopted by misinformation actors.

Then, we present the methods of the study, which was based on the features analysis of the online activities of 59 anti-vaccination actors. Our analytical contribution is through correspondence analysis—a common technique used to explore association between categorical variables. In our results, we show that anti-vaccination actors have both movement-like (membership dues and donations), celebrity-like (converting attention into money through advertising and donations), and junk news-like (advertising) material resource-gathering strategies that can be deployed interdependently in a hybrid process. These three strategies feed off each other, in turn producing a new logic, a hybrid monetization strategy, which is reflected by two public online activities of anti-vaxxers: simultaneously campaigning and capturing the public’s attention. We conclude by proposing “hybrid material resource mobilization strategies” as a theory that captures a key component of the interaction between anti-vaccination actors and today’s media systems.

## Theorizing the leveraging of harmful content online

In this section, we set a framework for understanding how misinformation content is leveraged on the Internet to gather material resources. In our approach, we focus on a sociological, group-based comparison and select group theories supported by the scholarship that discusses resource mobilization efforts, specifically those concerned with monetization. Anti-vaccination actors have features that relate them to other types of groups that monetize their efforts. The literature directed us to a classification based on anti-vaccination groups often being radical, some of their websites diffusing junk news, and the leaders sometimes acting similarly to online celebrities. Each model employs a slightly different blend of techniques for extracting resources but revolves around a key material resource—money—thus making content monetization a major template for gathering resources by entities like anti-vaccination actors. Hence, in the section, we compare anti-vaccination actors to the following three distinct models: radical social movements, online celebrities, and junk news sites.

### Radical social movements

One way we view the anti-vaccination community in this article is through the lens of “radical” social movement theory. This lens has been used to understand other harmful movements such as the Islamic State, white supremacists, and members of the alt-right (Phadke & Mitra, 2020; Sagramoso & Yarlykapov, 2021). The digital presence of such social movements can help them network and grow. For example, members of the alt-right have used social media and memes to spread their ideology to mainstream culture (Heikkilä, 2017). In several countries, radical right-wing movements organize through networks of online forums, which help create and share content that reinforces the overall radical message (Bright et al., 2020). Such movements are worth considering here because many of the anti-vaccination websites we study also organize themselves as an issue-oriented movement and encourage users to subscribe to a common collective effort. Some anti-vaccination groups are part of a broader phenomenon of conspiracy-driven movements that draw on a similar resource-gathering playbook, which includes harnessing platforms to spread their message at scale (Kata, 2012). There is also some commonality between anti-vaccination and other radical social movements in terms of the intensity of the emotions of “anger and betrayal [that] drive the growing connectedness” (Larson, 2020).

Monetization and funding in these social movements have been discussed using resource mobilization theory (RMT; Jenkins, 1983). RMT “is based on the notion that resources … are critical to the success of social movements” (Eltantawy & Wiest, 2011). Classic pre-Internet age RMT-focused studies emphasized the importance of elites in funding movements and argued that the role of the mass base in providing material support was minimal (Corrigall‐Brown, 2016). However, more recent studies have argued that the mass base might now play a more prominent role due to the digitization of movement operations (Johansson & Scaramuzzino, 2022). People and organizations that provide material resources to movements might also affect their operations, aims, as well as the degree of their radicalism (Cress & Snow, 1996). Hence, an ability to generate resources can affect the trajectory of a movement.

Material resources in the form of money have helped radical movements grow and transform in the past. White supremacist organizations were able to mobilize in part due to the revenue they generated. One approach to raise funds is to create the concept of membership and encourage people who believe in the cause to contribute money regularly. For example, organizing a white supremacist movement can raise “a maximum of a little over $40,000 a year” from membership dues (Anti-Defamation League, 2017, p. 3). White supremacists also hold conferences that can cost $100 or more per person to attend (Anti-Defamation League, 2017). In addition, movements such as these can mobilize resources by selling merchandise or information products like books or leaflets (Costanza-Chock, 2003; Van Aelst & Walgrave, 2002). RMT would suggest that these monetization efforts are then used to fund the activities of white supremacists, and there is evidence to back up this theory: the funds have been used for spreading propaganda or for legal defense costs (Anti-Defamation League, 2017).

It may be the case that anti-vaccination communities have similar material resource mobilization approaches. However, in the existing academic literature, it is uncertain what types of material resources are required by each movement type—and how these resources are gathered online (Cress & Snow, 1996). The scarce literature on resource mobilization by movements involved in socially divisive activities highlights how resource-plentiful organizations are more likely to make an impact than smaller groups that rely on weaker resource bases (Fetner & King, 2016).

### Online celebrities

Other literature we draw upon to understand the potential strategies for leveraging harmful content relates to alternative influencers or online celebrities. These individuals capture and maintain fame by broadcasting their activities on the Internet, often through video-sharing platforms such as YouTube (Khamis et al., 2017). They are “committed to deploying and maintaining one’s online identity as if it were a branded good, with the expectation that others also treat them like a branded good” (Senft, 2013). Online celebrities can campaign for their causes through testimony, advocacy, and their own style of content presentation (Tufekci, 2013). Some online celebrities support an anti-vaccination agenda, while anti-vaccination groups reportedly share other celebrities’ approaches to monetization (Featherstone et al., 2020).

Online celebrities turn followers’ attention into material resources, a process that is often referred to be part of the “attention economy” (Hendricks & Vestergaard, 2019; Tufekci, 2013). These individuals achieve attention by forming a network through content and self-branding techniques (Khamis et al., 2017). To capture audience attention, celebrities give an impression of perceived intimacy by interacting with their followers. The sense of closeness that celebrities contrive is central to their material success since their allure “is premised on the ways they engage with their followers to give the impression of exclusive, ‘intimate’ exchange,” and followers “are privy to what appears to be genuine, raw and usually inaccessible aspects of influencers’ personal lives” (Abidin, 2015). In the public health domain, perceived intimacy is critical for building connections and makes individuals look more trustworthy than, for instance, pharmaceutical companies (Donelly & Toscano, 2018). As a counterpoint to online harms, some studies have begun to investigate the benefits of collaborating with digital health influencers within niche communities to address vaccine hesitancy (Lutkenhaus et al., 2019). An online celebrity can leverage attention and intimacy for financial gain in a variety of ways, such as advertising, selling access to exclusive content, merchandise, or products (Alperstein, 2019; Geidner & D’Arcy, 2015). In a similar fashion to radical social movements, influencers may appeal for donations to keep their activities going, often asking for money through platforms such as Patreon (Brown & Hennis, 2019).

### Junk news websites

A final model for leveraging misleading content is junk news (Howard, 2020; Taylor et al., 2020). Junk news—a concept that includes news that is outright “fake,” but also news that is sensationalized, polarizing, or based on highly distorted versions of the facts—is a common feature of the contemporary media landscape (Bradshaw, 2019; Bradshaw & Howard, 2018; DiResta, 2018; Howard, 2020). Junk news producers own websites that are designed to appear like the website of a “traditional” news media organization, mimicking their layout and style, with a collection of headlines associated with pictures. However, the “news value” of the content itself is meager. This approach makes them somewhat parasitic on the reputation and authority generated by quality organizations (Ekman & Widholm, 2020). Junk news producers are well placed to compete with mainstream news outlets partly because their distribution and production costs are so low (Allcott & Gentzkow, 2017). A prominent characteristic of junk news is that it tends to generate higher interest and engagement than its “quality” counterparts (Bradshaw & Howard, 2018; Narayanan et al., 2018).

Along with spreading misinformation, junk news websites often attempt to mobilize material resources to sustain their operations or make a profit (Allcott & Gentzkow, 2017; Tokojima Machado et al., 2020). Indeed, there is good evidence that many junk news producers have a commercial motivation behind their activities (Braun & Eklund, 2019; Wardle & Derakhshan, 2017). The majority of commercial junk news producers are private actors, but even networks such as the widely discussed one that was maintained by the Russian state propaganda agency IRA during the US presidential election in 2016 attempted to monetize the content of their websites (Golovchenko et al., 2020). The major monetization strategy for this type of misleading content producer is the sale of third-party advertising space (Nizamani, 2020). Adverts often generate money when people click on them—a click on an advert normally generating an amount in the region of $0.15. As adverts are typically personalized and driven through extensive ad placement platforms such as Google AdSense, the main focus of junk news is to maximize traffic. As with online celebrities, junk news producers can also maintain a family of websites dedicated to the sales of products and services. The junk news websites themselves then act as a means of funneling attention to the linked commercially oriented websites via online adverts. As we discuss below, anti-vaccination actors often operate junk news websites, relying on advertising to monetize their content.

## Methods

We aim to understand how online anti-vaccination actors gather material resources. In this section, we describe our methodological approach. First, we manually selected a sample of 59 different anti-vaccination actors, each maintains at least one website (and often a family of associated social media profiles). Then, we conducted a multifaceted content and features analysis of each website and analyzed the types of material resource mobilization activity they deployed based on our review of online monetization methods. Finally, we conducted a linked sites analysis by examining the websites’ HTML code to determine whether the infrastructure of anti-vaccination actors was distributed across several coordinated domains. Our data collection and analysis took place between November 2020 and May 2021.

### Data and sampling

We built our sample of actors that spread anti-vaccination narratives from an existing actively curated list of misinformation websites,^1^ and supplemented this from official, media, and watchdog organization reports and academic research that are referenced in the review of the literature. In addition, we reviewed existing literature in the area, identifying possible websites maintained by anti-vaccination groups and adding them to our existing list of potential sites. We also followed links from the websites in this list and added new relevant websites, adopting a snowball approach to sampling. This helped create an initial directory of sources of vaccine misinformation in English.

We thematically coded the websites from the initial directory of sources to estimate their degree of anti-vaccination content. We analyzed the quantity and narratives of content related to vaccines present on each website. We counted content as anti-vaccination based on its credibility or bias about vaccines and on the presence of potentially misleading or harmful content and references to organizations that promoted such content. Every source was scored on a 3-point scale from 1 (“Almost exclusively anti-vaccination publications”) to 3 (“Few anti-vaccination publications”); see Appendix C in Supplementary Materials for details. This approach corresponded to a source categorization strategy adopted in previous research (Bradshaw et al., 2020).

Each website on our list of potential sources was coded twice, and reliability was measured through Krippendorff’s alpha (0.83). Following this procedure, we included in our final list only those websites which we categorized as “Almost exclusively anti-vaccination publications.” It is worth noting that we did not include large-scale misinformation operations which might occasionally push anti-vaccination narratives as part of various types of misleading content. In total, we selected 59 actors for inclusion. We provide sample characteristics in Appendix D in Supplementary Materials. We believe the resulting list contains the most significant English-language anti-vaccination websites in terms of the audience they reach and the scale of their operation.

## Analysis

During the next step, five researchers conducted content and features analysis (Kavada, 2012) on the websites in question in pairs, as well as any accompanying social media profiles. Almost two-thirds of the websites featured similarly branded social media accounts on platforms such as Facebook or Twitter. Our codebook is based on the monetization strategies suggested by our literature review above. The codebook drawn out of the three monetization styles of misleading content (radical social movements, online influencers, and junk news). We coded each website for the presence of the following features: appeals for monetary donations; sale of information products such as books and leaflets; sale of merchandise or other products; advertising banners; and options to become a “member” and thus pay membership dues. Table 1 shows which of these strategies are drawn from which of the monetization styles that we reviewed in the literature section. Appendix A in Supplementary Materials provides a more detailed overview of each strategy.

**Table 1.** Summary of strategies

	Radical social movement	Online celebrity	Junk news	Krippendorff’s alpha
Monetization strategy				
Appeal for donations	x	x		0.86
Sale of information products	x	x		0.61
Sale of merchandise/other products	x	x		0.61
Advertising		x	x	0.60
Membership dues	x			0.77
Other material resource mobilization				
Linked sites		x	x	NA

*Note.* See Appendix A in Supplementary Materials for a detailed description of the monetization strategies.

*Source:* Authors’ calculations based on data collected.

Coders looked at each of the narratives and website features and determined whether they were present or absent. Reliability was measured through Krippendorff’s alpha; all the reported variables had alpha above 0.6 (statistics per strategy are presented in Table 1). Lombard et al. (2010) state that these are appropriate levels for exploratory studies for such a measure. In cases where two coders disagreed, they produced the final code through discussion.

Previous studies suggest that actors involved in spreading misinformation rely on distinct but overlapping models of material resource mobilization. Table 1 demonstrates considerable overlap between radical social movements and online celebrities: both often appeal for donations and sell merchandise and information products. However, celebrities are more likely to focus on advertising, whereas radical social movements will ask for membership dues. Junk news organizations are distinct in focusing almost exclusively on (third-party) advertisement. In what follows below, we seek to locate anti-vaccination actors among this typology.

We further explored the 34 websites that monetized through donations to better understand what underlying infrastructures were being used to process payments. For this, we followed previous studies on using browser plug-ins WhatRuns and Wappalyzer that help navigate the back-end of a website (Au et al., 2020; Gerlitz & Helmond, 2013). We looked at each website in the sample to identify the underlying third-party services, such as PayPal, Donor Box, or Amazon Smile, being used to collect donations, and then confirmed the use of these third-party services by manually checking on the websites themselves.

As we highlighted above, one common feature of both the celebrity and junk news style of material resource mobilization is to maintain a family of linked websites, separating the ones that attract interest and attention from those which directly engage in monetization by selling products and services. To identify these connected websites, we used an automatic Google Analytics and AdSense identification (Google IDs) technique which has been applied previously to the study of junk content (Au et al., 2020). This allowed examining whether anti-vaccination actors distributed their resource mobilization infrastructure across several coordinated domains.

Our analysis of Google IDs relied on open data about Google Analytics and Google AdSense IDs, which are often present in the HTML code of a website, and which can be freely accessed. Google Analytics is a free service that provides website operators with data about who visits their websites. Website operators place a unique identification number on their pages to track information about their visitors’ identity (such as their location) or behavior (such as the amount of time spent on a page). More than 29 million websites used this free service to track visitors (Builtwith, 2020). Google AdSense is an advertisement platform that allows website owners to monetize their content by selling the space on their sites. Since a Google ID must be manually placed into the code of a website, these data can provide evidence of ownership or management of these websites. If the same ID is placed on different websites, this indicates at least some level of coordination.

Using a custom scraper developed for the task, we collected all Google IDs contained in the websites that we analyzed. We identified 28 domains from our sample that contained between them 31 Google Analytics IDs and two AdSense IDs. In order to discover linked websites, we compared these IDs to the database of SpyOnWeb (Hassan & Hijazi, 2018), a service that crawls Internet at large and scrapes Google IDs; that database contains approximately 12 million unique IDs. While this is only one way of examining what ad networks a website might use, Google IDs seemed to be the most prevalent architecture in our sample. For instance, we only found one website with an active Facebook Pixel link. We accept that this analysis is thus an imperfect approximation, but it helps provide insight into how reliant anti-vaccination actors are on Google’s ad services—the largest ad network in the world.

## Findings

We begin by presenting a breakdown of the monetization strategies of anti-vaccination actors, as outlined in Table 2. Of the websites we examined, the majority (85%) showed some evidence of monetization. Appeals for donations were the most common form of monetization, followed by sales of information products and merchandise/other products. Third-party advertising and membership dues were the least common forms of monetization. In addition, some actors maintained a family of linked websites, which we classified as another approach to material resource mobilization. We give more detailed examples of the types of monetization we found in what follows.

**Table 2.** Monetization strategies and other approaches to material resource mobilization

	Number of sites	Percentage
Monetization strategy		
Appeals for donations	34	58
Sale of information products	24	41
Sale of merchandise/other products	18	31
Advertising	13	22
Membership dues	10	17
Other material resource mobilization		
Linked sites	10	17
Total	59	

*Note.* Overall, only a few websites (eight) contained no evidence of a monetization strategy (alpha 0.89). Of the sites, 51 contained evidence of some form of the five monetization strategies or had linked websites.

*Source:* Authors’ calculations based on data collected.

### Appeals for donations

More than half of all the website owners monetized their content through donations (Table 2). Websites appealed for donations to support individuals negatively impacted by vaccines or for the distribution of anti-vaccination educational material, to fund the alternative medicine movement or just to keep the website going. The anti-vaccination actors engaged in this kind of behavior had the most in common with radical social movements.

Most websites had multiple methods of accepting donations. To better understand the material resource mobilization infrastructure used by the anti-vaccination actors, we examined the 34 websites that accepted donations to identify what third-party payment platforms were used. The results are presented in Table 3. The prevalent payment platform used by the sample of websites was PayPal, which allows easy transfers between individuals and organizations. In addition to this, we found 17 websites that accepted credit card payments through various third-party platforms, such as Stripe (five sites) and Donor Box (two), Benevity (one), most of them specializing in providing infrastructure for charities. Eleven websites noted they accepted more traditional forms of offline payment, such as checks, bank transfers, and money orders. Three websites accepted cryptocurrencies, which are often harder to trace than credit card transactions. Anti-vaccination websites also used novel online fundraising platforms to accept donations, including Amazon Smile, a donation platform tied to Amazon, Stock Donator, a service that allows stock options to be donated, and MyChange, a platform that rounds credit card purchases up to the nearest dollar and donates the leftovers. As we discuss below, some of these services are normally only available to organizations with official charitable status.

**Table 3.** Monetization infrastructure used to collect donations in the subset of websites that solicited donations (*n* = 34)

Payment platform	Number of websites	Percentage
PayPal	26	76.47
Credit card	17	50
Offline payments	11	32.35
Cryptocurrencies	3	8.82
Amazon Smile	3	8.82
Stock donator	2	5.88
Anchor.FM donations	1	2.94
Google Pay	1	2.94
MyChange	1	2.94

*Note.* Websites often had multiple channels to accept donations; this table presents a count of how often each payment/donation platform appeared in the sample.

*Source:* Authors’ calculations based on data collected.

### Information products, merchandise, and other products

More than one-third of the websites studied offered information or entertainment products for sale, such as books and films on anti-vaccination topics. They sold these products through their own websites or using third-party platforms. For instance, stopmandatoryvaccination.com maintained a list of books and films such as *Vaccine Epidemic: How Corporate Greed, Biased Science, and Coercive Government Threaten Our Human Rights, Our Health, and Our Children* ($10.29, Amazon). Meanwhile, a network of websites that include healthimpactnews.com and vaccineimpact.com sold such products as *Medical Kidnapping eBook*.

In addition to information products, almost one in three of the actors we analyzed was involved in e-commerce through selling either merchandise or other products such as natural health supplements. The promotion of health supplements is particularly widespread. For instance, stopmandatoryvaccination.com sold a product called “Pure Body Strength” that was advertised as a treatment for children who had been vaccinated and then developed health issues. Moreover, three of the websites specifically advertised medical care and treatment regimes. As discussed in detail below, anti-vaccination content producers were strongly linked to a network of 20 domains such as healthytraditions.com, vaccineimpact.com, or healthytraditions.com, parts of which were used to sell health supplements while other segments of the network promoted the anti-vaccination agenda. Thus, the owners of the websites linked their products to the core issue that they worked with.

### Advertising

One notable observation from our analysis was that only around one-fifth of the websites we surveyed displayed advertising banners, which allow for monetization through selling website space. The earnings are contingent on how many people click on a banner as well as overall web page visitor numbers. Some websites contained banners promoting anti-vaccination books and films or health supplements that can allegedly help tackle the supposed negative impacts of vaccination. For example, vaccines.news displayed multiple banner adverts for nutritional supplements and herbal remedies. These banners might also bring a percentage of sales to website owners as a direct marketing relationship (something which is common among the online celebrity community), though we are unable to make a definitive determination in the examples we have seen. Previous studies have found that the majority of advertisements placed on anti-vaccination websites emphasized vaccine harms (Jamison et al., 2020).

The website stopmandatoryvaccination.com is a notable example here because it explicitly listed the price of its advertising space. This website sold advertisement space starting at $59. The maximum advertisement cost was listed as $239, which would include four Facebook posts per month and an email ad. It is worth noting that, at the time of writing, Facebook has banned the stopmandatoryvaccination.com community: before this, the owners of the website claimed to be able to reach up to 130,000 members on this platform. In 2018, stopmandatoryvaccination.com had about 400,000 engagements on Facebook, with an individual post generating an average of around 7,000 engagements (Broderick, 2019).

### Membership dues

Of the actors we looked at, approximately one-sixth sought to collect funds by establishing a membership scheme on their websites. Many of these presented such schemes as an opportunity to provide regular support to the organization behind the website, thus becoming the patrons of an anti-vaccination community. Some also included the possibility for members to gain access to premium content. For example, greenmedinfo.com offered “Pro membership” for $89 per month, allowing users to access exclusive features of their platform. While some of the groups behind the websites stated that they were operating on an informal basis, it is worth noting that some of them had acquired official charitable status.

The anti-vaccination group Physicians for Informed Consent was a prominent example of an anti-vaccination community in our sample that relied on membership to mobilize material resources. The stated mission of this organization was to inform families about vaccines so that the family can make an informed decision. They also provided legal guidance and education on mandatory vaccines. Their main targets seem to be the measles and flu vaccines. In 2019, this organization was registered as a nonprofit in the U.S. It reported that its total earnings for that year were $165,188, which came from membership dues ($46,100) and donations/grants ($119,088). Membership dues for doctors were up to $350/year. Their website had a section allowing people to sign up to become a member to receive access to special features. As a registered charity, Physicians for Informed Consent was able to receive donations via Amazon Smile and Benevity. They also belonged to the Association of American Physicians and Surgeons, a group which has been criticized for promoting science misinformation, including the idea that there is a causal link between vaccines and autism (McInerney & Dougherty, 2020).

### Linked sites analysis

One of the dimensions of the associations we reveal relates to the links between anti-vaccination websites and other domains. Such links can point toward shared ownership or other strong ties. We used advertising and user analytics identifiers to trace the links between the sampled domains and websites beyond our sample. This helped identify several networks of linked sites. None of the domains from our sample belonged to more than one network. Two networks focused on spam content; thus, we did not include them in this analysis. Appendix B in Supplementary Materials provides an example of three clusters of the linked health-related sites with Google IDs that belonged to one of the sampled domains and were not mirrors.

First, the largest cluster that we analyzed linked twenty domains, including vaccineimpact.com—a website from our sample. Some of the domains were inactive. Most of the active domains were e-commerce websites such as healthytraditions.com that sold diet and nutrition supplements. For example, vaccineimpact.com featured a banner advertising a diet supplement product sold by healthytraditions.com. This points toward strong ties between at least some segments of this cluster. The link between sites such as vaccineimpact.com and this array of e-commerce websites suggests that anti-vaccination content might be used to promote and sell health supplements. The second cluster of five websites contained three active domains, including two that were not part of our original sample. These websites did not address vaccination issues but rather centered on self-development and health topics covered from an esoteric angle, including those focused on “new biology.” Third, our analysis revealed a link between two similarly branded and designed websites—thetruthaboutvaccines.com and thetruthaboutcancer.com. The latter was in our original sample and focused on vaccination content, while the former discussed broader health issues with an emphasis on cancer and “alternative” treatments.

### Correspondence analysis

In this section, we show how monetization strategies cluster together. We use correspondence analysis: a qualitative–comparative method frequently used to identify the association between categorical variables: anti-vaccination actors and monetization strategies (Clausen, 1998).

**Figure 1.**
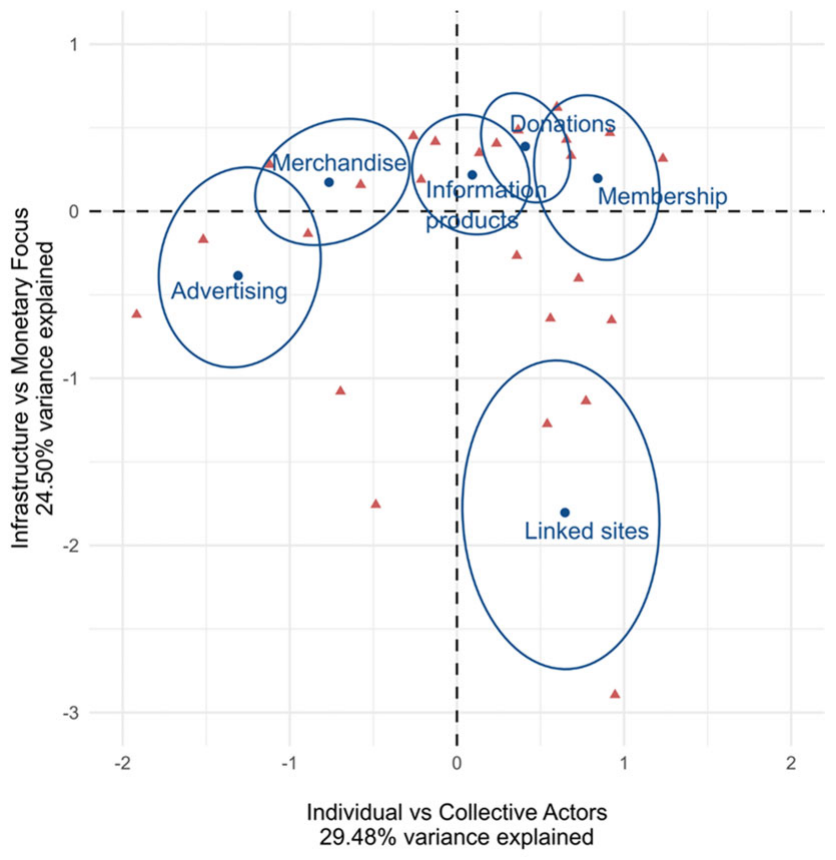
Correspondence analysis between different monetization strategies. *Note.* The ellipses represent 95% confidence intervals around the different strategies. *Source:* Authors’ calculations based on data collected.

The distribution of monetization strategies (and websites themselves) is shown in Figure 1, which plots their distribution in the top two dimensions identified by the correspondence analysis. We can interpret the results in the following way. The horizontal dimension represents a polarity between characteristics of anti-vaxx actors—from more personality-oriented figures that rely on advertising and selling branded merchandise to those that try to gather collectives and groups of supporters united around a cause. The vertical dimension represents the distinction between reliance on monetary instruments, such as cash or electronic funds, to reliance on infrastructure as a material resource, such as a network of sites. Together these dimensions account for 54% of the total variance in the dataset. The ellipses represent 95% confidence intervals around the different strategies; red points indicate individual anti-vaccination actors (Clausen, 1998). This analysis allowed us to identify three clusters of strategies that are typically found together: one which contains both advertising and the sale of merchandise; one which includes information products, donations, and membership; and one based solely around promoting linked websites.

### Limitations and future research

One important limitation of our approach is that we cannot estimate the volume of funds generated through each discussed strategy. However, in a few separate cases, we were able to estimate how much revenue was generated by websites based on their public tax returns, which we used as an illustration. Furthermore, while we studied the website and social media presence of anti-vaccination actors, we did not analyze the content of their email lists, which means we may only have an incomplete view of the types of resource-gathering activities they engage in. Finally, our Google ID data analysis was an imperfect proxy for the identification of linked websites. Google IDs are only one of the many services available. Moreover, there are potentials for false positives in this analysis. Some of the connections we identified may be spurious: for example, the websites may be created by the same website creation service or the same HTML code, along with Google IDs, reused to build a website, but not actually be managed by the same owner; although, as we shall show below, many of the connections we identified do appear to be genuine. More importantly, there is no requirement for individuals to use the same Google ID across multiple websites: and if they did not, we were not able to identify connections. Estimating the size of funds, investigating so far under-studied venues for resource gathering such as newsletters, and understanding the nature of links between the infrastructures of anti-vaccination actors are important avenues for further research.

Future studies should address these limitations by exploring other ways of revealing links between and beyond anti-vaccination communities, by attempting to better estimate media and economic effects of monetization strategies employed, and by analyzing relevant content in greater detail. Our three-model framework is just one of many ways scholars could approach these groups. Indeed, some other approaches might have enhanced our classification of anti-vaccination actors. For example, some people have psychological features that lead them to become active in anti-vaccination groups: they join these groups for reasons related to their rational and emotional decision-making as well as their political attitudes and life circumstances (Sobkowicz and Sobkowicz, 2021). This means that it might have been possible to compare the groups to political organizations or radical religious communities. To emphasize their political aspects, we could also distinguish the different forms of decision-making (authoritarian versus participatory) within groups, their ideological identification, or their approach to mobilization versus articulation (Melucci, 1996). The political links of these groups could be revealed by contextualizing their operation and tracing their political agenda through additional content analysis, interviews, and the computational analysis of comment sections. We could also assess group action outcome by tracing the levels of engagement with their content, assessing the possible income from their operation, or linking this to evolution in their audience’s opinions.

## Discussion

We investigated how anti-vaccination advocates build their information infrastructure by monetizing web content, and how their approach compares to existing models of material resource mobilization observed across other groups involved in spreading misleading or harmful information. In this way, our study contributed to the scarce research on the political economy of misinformation campaigns and their monetization systems. Our analysis demonstrated the diversity of resource mobilization strategies that issue-oriented misinformation actors employ—the financing that enables junk health news and anti-vaccination messaging to have broad reach. These strategies link them to the junk news industry, radical social movements, and online celebrity practices simultaneously.

We demonstrated that these models of resource mobilization are available to issue-oriented groups that circulate digital misinformation, such as anti-vaccination actors. Analysis showed that donations, sale of merchandise, and advertising were key monetization strategies. This was broadly consistent with reporting by watchdog organizations, as well as evidence published in academic studies that has analyzed other issue-oriented misinformation actors (Center for Countering Digital Hate, 2020; First Draft, 2021; Snyder et al., 2021; Tokojima Machado et al., 2020). However, it seems the role of advertising was overemphasized in previous reports, as donations are the most common financial strategy. This showed that misinformation actors can rely on a popular base in addition to or instead of elite or state funding.

In some ways, anti-vaccination campaigners operate like traditional social movements in their reliance on a popular base. However, just like many other contemporary movements, anti-vaccination actors have embraced more recent trends in movement-style organizing, often de-emphasizing traditional membership fees and forms of organization, preferring informal modes to official charity status, and, similar to online celebrities, emphasizing users’ attention along with easily available donation mechanisms (Herasimenka 2022; Tufekci, 2013). Just like junk news websites, some anti-vaxxers operate complex information flows of junk health news and analytical content to steer the audience, ultimately helping them wield noticeable communication power across more radical communities (Castells, 2011).

Anti-vaccination groups blend several media and social engagement strategies, taking full advantage of the back-end of information infrastructure to support their campaigns. Strategies include simply monetizing attention through traditional community membership mechanisms, but combining these with the modern digital affordances for networked content promotion and fluid agendas (Bennett & Segerberg, 2013). Such groups claim or mimic the status of being nonprofit charities or political action committees, while integrating with the ecosystem of cryptocurrencies. They offer services, collecting donations through paychecks for an offline consultation with one of their health consultants. This ability to blend newer logic to resource mobilization with the older logics of membership-based structures that collected yearly fees via paychecks suggests that anti-vaccination groups operated a hybrid logic of material resource mobilization. Chadwick (2013, p. 9) noted that hybridity “alerts us to the unusual things that happen when distinct entities come together to create something new that nevertheless has continuities with the old.” Concerning the use of the Internet, the concept of hybridity is often used to emphasize conflict and competition between older and newer logics of communication. This is directly relevant to the logic of operation that we observed in this study: people pursuing an anti-vaccination agenda, the origins of which date back to previous centuries, have blended the most innovative approaches to content monetization with the older logics of operation and funding, and this makes their material resource mobilization hybrid.

The key feature of this mode of material resource-gathering behavior is what we term hybrid monetization strategies. Movement, digital news, and celebrity monetization logics fed off each other, in turn producing updated approaches: hybrid monetization strategies which reflected in two key public online activities of anti-vaxxers—simultaneously building campaigning communities and capturing the attention of users to publicize and legitimize resistance to vaccination and public health programs on COVID-19. This might constitute a key element of the communicative context, organizational interactions, and political outcomes of misinformation production by these groups.

Our theory reflects that misinformation actors operate in what Chadwick (2013) has called a hybrid media system—a space where the logics of organizational, technological, and social media systems mix. We contribute with a better understanding of structural distortions to this system (Chadwick & Stanyer, 2022). In previous ground-breaking studies, scholars have shown how digital platforms have been incorporated by and, in turn, been transformative for organizations communicating prominent issues (Bennett & Segerberg, 2013; Earl & Kimport, 2011; Howard, 2005; Karpf, 2012; Kreiss, 2012), leading to the emergence of “highly networked modes of quasi-organization that trade in obfuscation on key issues” (Chadwick & Stanyer, 2022, p. 8). The most important factor that often prevents us from analyzing these quasi-organizations seems to be their obscure qualities. This obscurity leads to the perception of the communicative context of misinformation production as either large elite-driven operations, hence reflecting the legacy view of propaganda (Jowett & O′Donnell, 2011), or loose assemblages of co-production networks without visible external incentives (Mirbabaie et al., 2022), which is in line with connective action theory (Bennett & Segerberg, 2013).

The hybrid material resource mobilization strategies framework advances the theory of this quasi-organizing for the communicative context of misinformation production. It points out that when hybrid mobilization strategies are present, we are likely to be dealing with something more organized, focused, and intentional than the “widespread, irrational social effects” of “unidirectional propaganda”—a view that currently constitutes one of the foundations for much of the research on misinformation (Anderson, 2021, p. 52). We argue that anti-vaccination groups, like many other evolving organizations, have embraced this transformation to enhance their organizational capabilities when working with their resources. Rather than creating an alternative social space of “connective action” (Bennett & Segerberg, 2013), they incorporate traditional propaganda (advertising) or movements (donations) monetization strategies along with newer forms that seem to be more innovative, such as the digital celebrity model.

The organizational interactions that we observed signal that misinformation actors may develop what Reese and Chen (2022) describe as hybrid networks or institutions. These hybrid institutions bridge groups that practice traditional media approaches and largely anonymous citizens who are linked through shared ethos and tools. The ethos of the mistrust of vaccination programs bridges such institutions while tools for monetization make them more sustainable.

We have demonstrated that groups spreading misleading content to mobilize citizens for political goals, such as those linked to QAnon theories, cannot be viewed simply as loosely linked networked groups that are united by a common discourse. They also exhibit the properties of celebrity fans, while the clusters of their infrastructures can act like a news organization. Vice versa, junk news organizations like Natural News might contain the elements of a movement with its followers inspired by similar cultural processes and open to similar mobilization mechanisms as those exhibited in movements. This discussion requires further investigation of a complex digital misinformation landscape. The concept of hybrid material resource mobilization allows a clearer understanding of the infrastructural conditions under which the misinformation industry operates. It also enables a better-suited public intervention into this problem. The fundamental premise of this intervention should be an awareness that issue-oriented misinformation actors can be hindered by making it more complex for them to gather material resources. Classic resource mobilization-focused studies have overemphasized the role of funding as a type of resource that actors seek to control (Cress & Snow, 1996). Our findings suggest that digital infrastructures are at least as important as the message, especially for issue-oriented campaigning that goes against scientific consensus and public health guidelines. In this way, access to digital information infrastructures is a vital resource for such campaigns.

## Conclusion

Preventing harmful actors, like anti-vaccination groups that spread misinformation, from mobilizing material resources could hinder their expansion, though it is hard to assess the impact of such interventions. Watchdog organizations suggest that anti-vaccination industry operates large budgets linked to the discussed resource mobilization strategies (Center for Countering Digital Hate, 2020). Digital platforms have introduced policies of removing misleading and harmful content and their producers’ accounts (Herasimenka et al., 2022). However, some of the key resource mobilization strategies we discuss seem to be not captured by these policies.

Indeed, some of them, such as seeking donations and membership fees or selling merchandise, are harder to address. Moreover, monetization is only one element of the larger ecosystem sustaining anti-vaccination groups. As seen here, the plethora of back-end mechanisms, including payment intermediaries, donation collection services, and storefront support, plays an important role. Tackling the larger anti-vaccination ecosystem will thus require a more concerted effort to undermine it. For example, platforms like PayPal and Google should not accept donation, membership, or merchandise payments from known anti-vaccination entities; Amazon should consider removing products that are used to disseminate vaccine misinformation; crowdfunding websites should not host anti-vaccination campaigns; and platforms should tackle advertising that helps fund them.

Some have argued that managing digital resources does not require any funding and hardly any effort, attention, or time (Shirky, 2008). This thinking was related to the argument about the availability of Internet affordances: the Internet made mobilization of all types of resources easier to achieve compared to previous decades by dramatically reducing the costs of movement operation (Earl & Kimport, 2011). The ambiguity of the concept of “resources” added to the confusion (Cress & Snow, 1996). However, more nuanced studies show that all types of actors became dependent, including in their material resource mobilization strategies, on a newer type of resource: access to digital platforms (Johansson & Scaramuzzino, 2022). Deplatforming, for example, causes serious distress to these communities. In other words, “the ability to marshal resources still matters” (Hestres, 2017). Recent efforts by technology companies have focused on deplatforming anti-vaccination actors, but we see that these actors still exist on their own websites, which they are using to gain financial incentives. We hope this article helps policymakers understand the diverse ecosystem and bring to light the other ways in which harmful communities sustain their digital presence: through material resource mobilization.

## Supplementary Material

jqac043_Supplementary_Data

## Data Availability

The data underlying this article are available in the article and in its online supplementary material.

## Footnotes

1 We relied on a list of general misinformation sources published in Bradshaw et al. (2020). We updated it regularly and used it to build our sample with a specific focus on misinformation about vaccination programs.
