# Supplementary material for: The political economy of digital profiteering: communication resource mobilization by anti-vaccination actors
Source: J Commun. 2022 Dec 24;73(2):126–37. doi: 10.1093/joc/jqac043 (PMC10066223; doi:10.1093/joc/jqac043)
Supplement: jqac043_Supplementary_Data [file jqac043_supplementary_data.docx]

Supplemental materials document

## Appendix A. Monetization Strategies Coding Scheme.

Appeal for donations: Whether the online presence appealed for any kind of donation. We also looked at the payment platform, if donations were available.

Sale of information products: Whether books, leaflets, videos, audio recordings, or other information products were for sale on the website. This did not include third-party advertising for such materials.

Sale of merchandise or other products: Whether merchandise or other products were directly for sale on the website. Merchandise might include branded clothing or other types of branded goods. Other products often included health supplements.

Advertising: Whether the online presence of the anti-vaccination actor carried banner adverts placed by third-party advertising networks such as Google AdSense. Direct adverts for products or services directly associated with the actor themselves, for example, partnerships with health food stores, also count in this strategy.

Membership dues: Whether the online presence had a concept of membership, or asked people to make a recurring contribution.

## Appendix B. Examples of Google ID Data Analysis.

| 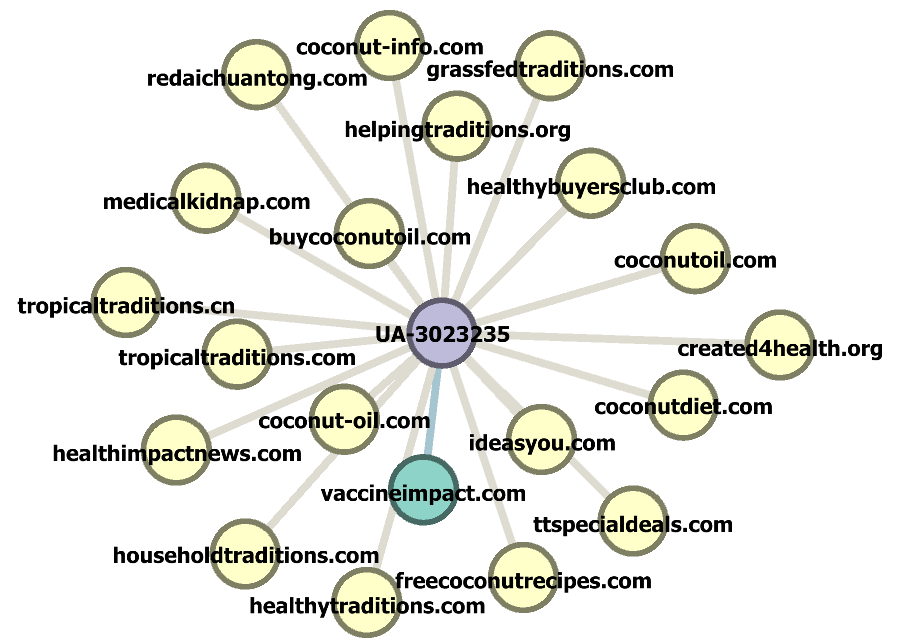1 |
| --- |
| 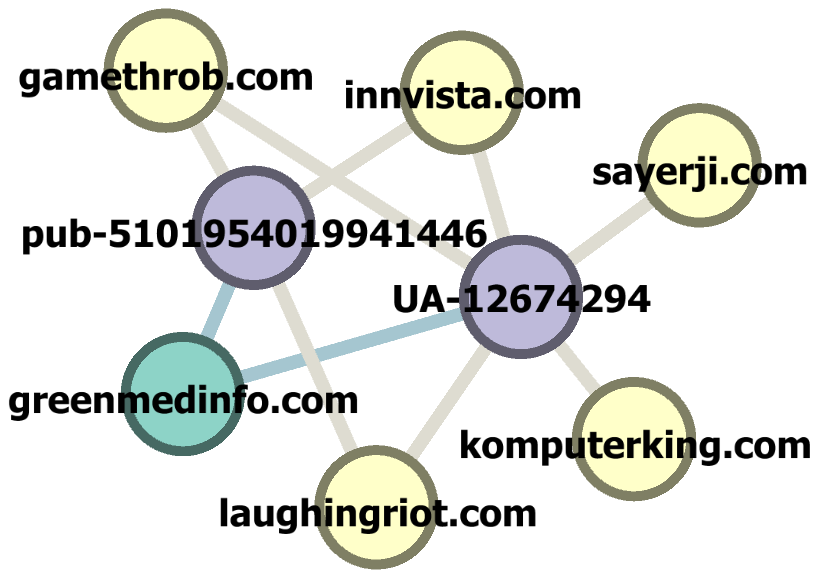2 |
| 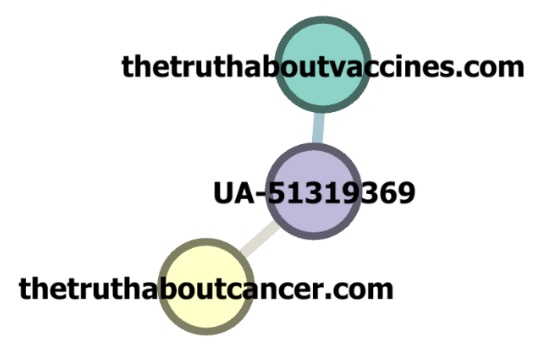3 |

Note. Domains from the original sample are colored with green. Google ID nodes are colored purple, yellow nodes are next nodes in the shared information infrastructure.

## Appendix C. Sample Coding Scheme.

1. Website - a collection of related and linked web pages hosted under a single domain name. Include “third” domain websites hosted on platforms such as WordPress.
2. Anti-vaccination narratives present: This website deliberately publishes or aggregates misleading, deceptive or incorrect information purporting to be real information about vaccination. To be classified as such, a website must fulfill at least three of the following criteria:

Credibility: Websites rely on false information and conspiracy theories, which they often employ strategically. Their sources are often untrustworthy.

Bias: Information present is highly biased, and content frequently includes strongly opinionated commentary and inflammatory viewpoints.

Counterfeit: These websites mimic established news reporting. They counterfeit fonts, branding, and stylistic content strategies. Commentary and content are stylistically disguised as news, with references to news agencies and credible sources, and headlines are written in a news tone with date, time, and location stamps.

Style: These websites use emotionally driven language with emotive expressions, hyperbole, ad hominem attacks, misleading headlines, excessive capitalization, unsafe generalizations and logical fallacies, and moving images.

References: References to established anti-vaccination groups are identified in the academic literature.

1. Degree of anti-vaccination content present (a three-point scale): If a website had any publications that employed anti-vaccination narratives, assess the scale of the prevalence of these narratives using a three-point scale from 1 (“Almost exclusively anti-vaccination publications”) to 3 (“Few anti-vaccination publications”). Provide examples.

## Appendix D. Sample Characteristics.

|  | | Number of websites | | | |
| --- | --- | --- | --- | --- | --- |
| **Top Level Domain**  com | | 29 | | | |
| org | | 18 | | | |
| [co.uk](http://co.uk) | | 3 | | | |
| [org.uk](http://org.uk) | | 3 | | | |
| Other | | 6 | | | |
| **WHOIS Registrant by Country** | |  | | | |
| US | | 36 | | | |
| UK | | 8 | | | |
| N/A | | 6 | | | |
| Canada | | 4 | | | |
| Switzerland | | 2 | | | |
| Iceland | | 2 | | | |
| India | | 1 | | | |
| **Purpose of Site** |  | | |  | |
| Health news/information |  | | 43 | |  |
| Campaigning |  | | 27 | |  |
| Other |  | | 12 | |  |
| Shop/e-commerce |  | | 12 | |  |
| General news (beyond health) |  | | 11 | |  |
| Celebrity/one-person website |  | | 7 | |  |
| Discussion/maintenance of community |  | | 5 | |  |
| Brand/product promotion/building |  | | 5 | |  |
| Doctor consulting |  | | 3 | |  |

Source: Authors’ calculations based on data collected, WHOIS data, and content analysis.
